# Iron treatment of pregnant sows in a Danish herd without iron deficiency anemia did not improve sow and piglet hematology or stillbirth rate

**DOI:** 10.1186/s13028-019-0497-6

**Published:** 2019-12-09

**Authors:** Sheeva Bhattarai, Tore Framstad, Jens Peter Nielsen

**Affiliations:** 1 Department of Veterinary and Animal Sciences, Faculty of Health and Medical Sciences, University of Copenhagen, Grønnegårdsvej 2, 1870 Frederiksberg C, Denmark; 2 Department of Production Animal Clinical Sciences, Faculty of Veterinary Medicine, Norwegian University of Life Sciences, Campus Adamstuen, 0033 Oslo, Norway

**Keywords:** Anemia, Hemoglobin, Iron deficiency, Iron injection, Pig, Pregnancy

## Abstract

**Background:**

Anemia characterized by low hemoglobin concentration (HbC) is common in indoor housed pregnant sows. Iron is essential for hemoglobin synthesis and a number of metabolic processes including DNA synthesis and regulation of enzyme systems. In sows, anemia has been linked to lower HbC in piglets and increased occurrence of stillbirths. Therefore, the main objective of this study was to evaluate the effect of iron injection on hematology of pregnant sows and their offspring. Other objectives were to evaluate the effect of this injection on the probability of stillbirths and to study the tolerability of injected iron.

**Results:**

A sow herd with bi-weekly batch farrowing was selected for the study and 100 sows at mid-gestation were randomly assigned to either a treatment (FeT) or a control (FeC) group. At the time of recruitment to the study (baseline), 46% of the sows in the herd were anemic with a HbC less than 103 g/L. However, none of the anemic sows had iron deficiency anemia on erythrocyte characterization. HbC decreased numerically during gestation in both the FeT (− 2.48 g/L) and FeC (− 2.99 g/L) groups but the decrease was insignificant between the groups (P = 0.79). Likewise, the change from baseline to farrowing and from baseline to post-farrowing in other hematologic variables was similar for both groups. The percentage of transferrin saturation was not statistically different between groups (P = 0.14). There was a batch effect (week of breeding) in most of the hematologic variables. The probability of stillbirth in the two groups did not differ (P = 0.94). None of the hematologic variables in piglets was significantly different between the two groups. The sows tolerated the iron injection well.

**Conclusions:**

Intramuscular injection of two doses of 2500 mg iron 2 weeks apart at mid-gestation did neither change hematologic variables in sows nor in the piglets at farrowing. Similarly, iron treatment did not reduce the probability of stillbirths among the offspring. The sows recruited in this study tolerated the iron injections well. Further characterization of erythrocytes did not support that sows had iron deficiency anemia at baseline. Therefore, further studies on animals with well-defined anemia and with focus on the iron regulating hormone hepcidin are recommended.

## Background

Anemia characterized by low hemoglobin concentration (HbC) in sows is often encountered in intensive sow production herds [1]. Iron is required for the synthesis of hemoglobin (Hb) and in many processes maintaining normal structure and function of cells [2]. Iron requirement grows rapidly during late pregnancy in sows when they increase their erythrocyte mass and prioritize the iron supply to the developing fetuses [3, 4]. The current recommendation of iron supplementation in dry feed for pregnant sows is 80 mg/kg [5]; a level that has not been revised for four decades [6]. Some attempts have been made to increase the maternal iron supplies by oral supplementation but without significant effect on hematologic variables [7–11]. It is therefore possible that the current in feed iron supplementation strategy does not provide a sufficient iron uptake to meet the iron requirement of present day hyper-prolific sows.

The level of stillbirths of piglets has increased in Denmark [12] and worldwide [13] simultaneously with the selection of sows with greater litter sizes. Sow HbC is reported to be one of the risk factors for stillbirths [14]. We have recently shown that an increased stillbirth rate may be associated with low HbC of the sow at farrowing [15]. Adequate iron supplementation to the pregnant sows is expected to improve HbC in the sow and offspring and decrease the prevalence of stillborn piglets.

Uptake of oral iron is not always consistent [16] and may be limited by e.g. antagonism among minerals [17]. Parenteral iron treatment of anemia in pregnant sows may therefore be a better alternative.

The primary objective of this study was to evaluate the effect of iron injection on hematology of pregnant sows and the offspring piglets. Other objectives were to evaluate the effect of iron injection in pregnant sows on the probability of stillbirths and to study the tolerability of iron injection.

## Methods

### Study design

The study was a randomized clinical trial and was approved by the Danish Animal Experiments Inspectorate and the Danish Medical Agency (Approval number 2015-15-0201-00522). The study was carried out between May and September 2015.

### Setting

#### Herd selection procedure

A cross-sectional study was carried out in five herds in Denmark including 248 sows at mid-gestation in order to select a herd with low level of HbC for the clinical trial. From the cross-sectional study, the herd with lowest average HbC and highest prevalence of anemic sows was chosen for the clinical trial. The cut off value for anemia was chosen as 100 g/L [8].

#### Herd description

The herd selected for the clinical trial was located in the eastern part of Denmark. The herd had approximately 1000 sows (Landrace × Yorkshire, Dan Avl company) with a herd average of 1.8 stillborn piglets and 17 live born piglets per litter. The herd had a Specific Pathogen Free (SPF) status i.e., absence of infection with *Sarcoptes scabiei*, *Haematopinus suis*, *Brachyspira hyodysenteriae*, toxin producing *Pasteurella multocida*, *Actinobacillus pleuropneumonia* serotypes 1–10 and 12, *Mycoplasma hyoneumoniae* and porcine reproductive and respiratory syndrome virus. The sows were kept in separate stalls between weaning and until 2 weeks after insemination. Hereafter, sows were loose-housed throughout the gestation in small stalls. A week before expected date of farrowing, the sows were moved to individual farrowing crates. The gestation groups had automatic electronic feeders where the sows could be individually monitored for feeding. Water was provided ad libitum. Iron was supplemented in the dry feed at 83 mg/kg. The herd was regularly monitored by a herd veterinarian and had no diagnosed infectious diseases or conditions affecting herd productivity. The selected sows were apparently healthy on inspection on the days of blood sampling or interventions.

### Identification of sows

All sows at day 50–62 of gestation were listed and first parity sows were excluded as studies indicate higher HbC in such sows [18, 19]. The sows belonged to two bi-weekly batches (week of breeding); 77 sows from first batch and 76 from the second. Routine management and feeding was similar in both of the batches. Blood sampling was done before the commencement of the trial for sow selection. For the clinical trial, 50 sows within each batch with lowest HbC were chosen. The HbC in sows ranged from 85.38 to 119.21 g/L. Randomisation of sows into control and treated groups was done using random number generator in Microsoft excel.

### Interventions

Between days (d) 56–69 of gestation, sows in the treated group (FeT) were injected with 12.5 mL (2500 mg elemental iron, Fe III) of iron dextran (Uniferon^®^, Pharmacosmos, Denmark) intramuscularly in the neck. Uniferon® contains 200 mg dextran bound iron and is approved for the prevention and treatment of iron deficiency anemia in piglets. Sows in the control group (FeC) were injected with 12.5 mL of isotonic saline intramuscularly in the neck on the same day. The volume of injected material was divided roughly into two equal parts and injected on each side of the neck to reduce discomfort from the volume injected. Same doses were given again after 2 weeks, i.e., between day 70 and 84 of gestation.

No interference in the farm routine management was made during the study. Farmer and farmworkers were blinded to the groups.

### Blood samplings and hematology

Blood from the sows was sampled three times. First sampling was done a week before the first treatment (i.e., around mid-gestation). Second sampling was done between 3 and 22 days before farrowing and the last sampling was done between 19 and 36 days after farrowing.

The sows were restrained using a snare and blood was withdrawn into each of 10 mL EDTA (BD Vacutainer, K2E 18.0 mg, BD, Plymouth, UK) and plain (BD Vacutainer CAT (Clot Activator Tube, BD, Plymouth, UK) tubes from the jugular vein using 18 G × 1½  in. needles (Venoject, Terumo Europe N.V., Leuven, Belgium). The EDTA samples were taken on all three blood sampling occasions whereas plain samples were taken only at second sampling. The EDTA samples were stored at 4 °C and analysed within 2 days of collection, while the separated serum samples were frozen (− 20 °C) until analysis. The EDTA blood samples from sows were analysed for Hb concentration, erythrocyte count (RBC), hematocrit (HCT), mean cell volume (MCV), mean cell hemoglobin (MCH), mean cell Hb concentration (MCHC), red blood cell distribution width (RDW) and Hb distribution width (HDW). The values of HbC obtained from the laboratory were multiplied by 16.11 to convert from mmol/L to g/L [20]. The serum samples from the second sampling were analysed for serum iron (SI) and total iron-binding capacity (TIBC). Transferrin saturation (TSAT) was calculated using the formula: TSAT (%) = (serum iron ÷ TIBC) × 100. All hematologic testing was performed using the Advia 2120i Hematology System (Siemens Healthcare Diagnostics Inc, Tarrytown, New York), while SI and TIBC were tested using the Advia 1800 Chemistry System (Siemens Healthcare Diagnostics Inc) at the Veterinary Diagnostic Laboratory, Department of Veterinary Clinical Sciences, University of Copenhagen.

### Sampling of piglets

Blood sample was taken from two stillborn piglets per litter at most. These two piglets were selected based on convenience in case of many stillborns. From each litter, two liveborn piglets were blood sampled before colostrum intake. When pre-colostrum piglets were not available, post-colostrum piglets were sampled. In case of stillborn piglets, blood was directly withdrawn from the heart and *vena cava* during necropsy while for liveborn piglets; blood was withdrawn from the anterior *vena cava*. Blood was withdrawn into 5 mL EDTA tubes and subjected to complete hematology as described for sows. Additionally, reticulocyte indices were also determined in piglets, which included reticulocyte count (absolute and relative), mean reticulocyte cellular volume (MCVr), reticulocyte red cell distribution width (RDWr), mean reticulocyte cell Hb concentration (CHCMr), reticulocyte Hb distribution width (HDWr) and reticulocyte Hb content (Chr). SI and TIBC were not measured in piglets.

### Identification of stillborn piglets

At farrowing, all dead piglets were collected and marked with sow number. The dead piglets were necropsied and stillborn piglets were verified using a lung flotation test. Mummified foetuses were recorded, but not included in the trial.

### Clinical and other recordings

For each sow the following recordings were made: parity of the sow, day of gestation, expected date of farrowing, number of stillborn piglets, number of live born piglets, number of mummified piglets and number of total born piglets. General inspection of the sows for obvious disease was made. In order to study the tolerability of injected iron, the injected sites were checked for obvious swelling, redness, or lesions after the first injection. The farmer was asked to notify any conditions related to the injections. No deep palpation or tissue biopsies were done.

### Statistical analysis

Data analysis was performed using SAS 9.4 (SAS 9.4, SAS institute Inc, Cary, NC, USA). Two parity categories of the sows were defined; parity rank 1 which included second and third parity sows, and parity rank 2 which included sows with parities higher than three.

Sows were divided into two groups; anemic with HbC < 103 g/L and non-anemic with HbC ≥ 103 g/L. Anemia in sows was categorized morphologically into three categories: microcytic (MCV < 57 fL), normocytic (MCV ≥ 57 ≤ 69.3 fL) and macrocytic (MCV > 69.3 fL). It was further characterized as hypochromic (MCHC < 18.37 mmol/L), normochromic (MCHC ≥ 18.37 ≤ 20.31 mmol/L) and hyperchromic (MCHC > 20.31 mmol/L). These cut off values were chosen based on our recent study on reference intervals for Danish sows at mid-gestation [21]. A sow was considered to be suffering from iron deficiency anemia (IDA) when HbC, MCV and MCHC values were below the lower limits of all these reference intervals.

The hematologic and biochemical variables of the sows were analysed in separate models using a general linear model (PROC GLM procedure) with experimental group, parity rank and batch as explanatory variables. In each case, the mean change in those variables compared to the baseline was used as the outcome variable. The differences in hematologic variables of piglets between groups were tested individually using linear mixed model (PROC MIXED procedure) in separate models for each category of piglet. Sow was considered as a random variable during each analysis with sex of the piglet and parity rank of the sow as other explanatory variables. The variables were removed from each model using backward elimination. Experimental group was forced in each model as it was the main predictor of interest. The normality of residuals of GLM and MIXED model was examined using QQ plot and histograms. In case of non-normal residuals for piglet data, data was normalized using either log, square root or exponential transformations. For pre-colostrum piglets, percentage of reticulocytes and HDWr were normalized and for stillborn piglets, RBC, MCH, HDW and number of reticulocytes were normalized. A generalized linear mixed model was fitted (PROC GLIMMIX procedure) for those variables which could not be normalized by transformations. RDW for pre-colostrum piglets, RDW, RDWr and HDWr for post-colostrum piglets and MCV, MCHC, RDW, percentage of reticulocytes and MCVr for stillborn piglets could not be normalized by transformation.

A generalised linear model (PROC LOGISTIC procedure) was fitted to analyse the differences in the probability of stillbirth between the two groups. The explanatory variables in each of the analyses were experimental group, parity rank of the sow, total number of piglets born and their interaction.

Statistical significance was set at P < 0.05 for all the tests.

## Results

Altogether 100 sows were included in the study; however, two sows were lost for follow-up in subsequent blood samplings. The sows belonged to parities between two and seven. In the FeC group, 22 sows belonged to parity rank 1 and 28 sows belonged to parity rank 2 whereas in the FeT group, 30 and 20 sows belonged to parity rank 1 and parity rank 2, respectively.

In the first 12 h after farrowing, 197 piglets from the 100 litters were sampled, out of which 107 were males, 89 were females and one was unidentified. Blood sampling was done from 62 pre-colostrum piglets, 51 post-colostrum piglets and 84 stillborn piglets.

### Anemia in sows

Altogether 46 sows were anemic at baseline; 23 in each of the FeT and FeC group. None of the sows in either group had microcytic erythrocytes. In FeT group, 46 sows had normocytic erythrocytes while 4 had macrocytic erythrocytes. In FeC group, 49 had normocytic erythrocytes and only one had macrocytic erythrocytes. The number of sows with hypochromic and normochromic erythrocytes was 10 and 40 in FeT group whereas in FeC group, the numbers were 6 and 44, respectively. There were no sows with hyperchromic erythrocytes. None of the anemic sows had IDA.

### Hematologic variables in sows

The hematologic variables of the sows in the two groups at the three sampling points and the mean change compared to the baseline are presented in Table 1. There was no significant change in any of the hematologic variables between the two groups (Table 1). Batch was statistically significant in most of the models.

**Table 1:** Effects of iron treatment during gestation on hematologic variables of sows

	Baseline		Post-treatment		Change (post-treatment-baseline)		P-value	Post-farrowing		Change (post-farrowing-baseline)		P-value
	FeC	FeT	FeC	FeT	FeC	FeT		FeC	FeT	FeC	FeT	
HbC, g/L	103.81 (7.14)	103.65 (7.31)	100.90 (7.46)	101.17 (6.70)	− 2.99	− 2.48	0.79	100.96 (7.94)	101.46 (6.84)	− 2.95	− 1.93	0.59
RBC count, 10^12^/L	5.30 (0.41)	5.33 (0.43)	5.38 (0.40)	5.47 (0.49)	0.09	0.14	0.58	4.85 (0.40)	4.88 (0.39)	− 0.45	− 0.43	0.82
HCT, L/L	0.34 (0.02)	0.34 (0.02)	0.35 (0.02)	0.35 (0.02)	0.01	0.01	0.70	0.31 (0.02)	0.31 (0.02)	− 0.02	− 0.02	0.73
MCV, fL	64.71 (2.45)	64.35 (2.64)	65.75 (2.60)	65.23 (3.04)	0.92	0.88	0.88	65.00 (2.29)	64.68 (2.92)	0.27	0.32	0.87
MCH, fmol	1.21 (0.05)	1.20 (0.05)	1.16 (0.04)	1.15 (0.05)	− 0.05	− 0.05	0.68	1.29 (0.05)	1.29 (0.06)	0.07	0.08	0.76
MCHC, mmol/L	18.77 (0.39)	18.77 (0.43)	17.67 (0.43)	17.63 (0.36)	− 1.10	− 1.13	0.69	19.91 (0.48)	19.94 (0.55)	1.14	1.16	0.90
RDW, %	15.67 (0.69)	18.85 (0.92)	17.13 (0.64)	17.41(1.18)	1.48	1.55	0.21	16.01 (0.67)	16.15 (1.14)	0.32	0.30	0.88
HDW, mmol/L	0.98 (0.07)	1.01 (0.14)	1.03 (0.05)	1.08 (0.17)	0.05	0.06	0.28	1.09 (0.06)	1.11 (0.16)	0.11	0.10	0.51

Values are mean (standard deviation), *FeC* control group, *FeT* iron treated group, *HbC* hemoglobin concentration, *RBC* red blood cell, *HCT* hematocrit, *MCV* mean cell volume, *MCH* mean cell hemoglobin, *MCHC* mean cell hemoglobin concentration, *RDW* red blood cell distribution width, *HDW* hemoglobin distribution width

### Biochemical variables in sows

Biochemical parameters were measured only from second blood sampling of sows. The mean SI concentration in FeT group (18.57 ± 3.51 µmol/L) was significantly lower compared to FeC group (20.78 ± 3.81 µmol/L) (P = 0.003). Similarly, TIBC was significantly lower in FeT group (67.33 ± 5.33 µmol/L) compared to the FeC group (71.63 ± 5.22 µmol/L) (P < 0.0001). Batch was also significant in this case (P = 0.02). There was no difference in TSAT between FeT (27.58 ± 4.95%) and FeC (29.03 ± 4.91%) groups (P = 0.14).

### Prevalence of stillbirths

On an average, FeT sows had slightly fewer (6.6%) stillborn piglets compared to 7.1% in FeC sows. FeT sows had 17.61 total born piglets while FeC sows had 18.53 on an average. On logistic analysis, the probability of stillbirth in the two groups did not differ significantly (P = 0.94) nor there was a significant effect of total born piglets on stillbirth probability (P = 0.63).

### Hematologic variables and body weight in piglets

None of the hematologic variables in piglets was significantly different between the two groups (Table 2). Likewise, the body weight of the piglets did not differ between the groups (Table 2).

**Table 2:** Weight and hematologic variables in piglets born from two experimental groups of sows

Piglet type	− Colostrum				P-value	+ Colostrum				P-value	Stillborn				P-value
Group	FeC		FeT			FeC		FeT			FeC		FeT		
	N	Mean (SD)	N	Mean (SD)		N	Mean (SD)	N	Mean (SD)		N	Mean (SD	N	Mean (SD)	
Weight, g	20	1450.55 (300.65)	42	1355.90 (298.44)	0.24	28	1417.29 (245.62)	23	1492.91 (312.88)	0.43	43	1077.16 (355.56)	41	1076.00 (436.25)	0.86
HbC, g/L	20	115.42 (7.84)	42	109.08 (14.34)	0.07	28	98.21 (18.76)	23	91.54 (14.60)	0.08	43	100.81 (24.04)	41	98.74 (23.36)	0.82
RBC count, 10^12^/L	20	5.85 (0.56)	42	5.57 (0.84)	0.23	28	4.94 (0.81)	23	4.71 (0.83)	0.21	43	4.08 (1.49)	41	4.06 (1.44)	0.93
HCT, L/L	20	0.39 (0.03)	42	0.38 (0.04)	0.39	28	0.33 (0.05)	23	0.31 (0.05)	0.09	43	0.34 (0.12)	40	0.34 (0.11)	0.87
MCV, fL	20	68.48 (3.94)	42	70.23 (4.88)	0.24	28	67.46 (3.66)	23	66.25 (4.28)	0.45	43	82.15 (7.81)	40	83.20 (8.04)	0.59
MCH, fmol	20	1.22 (0.10)	42	1.22 (0.10)	0.79	28	1.23 (0.08)	23	1.21 (0.09)	0.73	40	1.52 (0.42)	39	1.46 (0.28)	0.45
MCHC, mmol/L	20	17.95 (1.09)	42	17.41 (1.02)	0.12	28	18.22 (0.88)	23	18.31 (0.94)	0.65	40	18.42 (6.74)	39	17.42 (3.12)	0.34
RDW, %	20	16.04 (16.04)	42	16.38 (0.91)	0.75	28	17.26 (2.50)	23	16.86 (1.81)	0.73	43	17.63 (4.96)	40	16.81 (3.19)	0.37
HDW, mmol/L	20	1.97 (0.24)	42	2.03 (0.22)	0.70	28	1.92 (0.21)	23	1.92 (0.16)	0.87	43	1.46 (0.53)	40	1.51 (0.35)	0.40
Reticulocytes, 10^9^/L	20	254.99 (57.10)	42	258.24 (73.01)	0.83	28	235.14 (64.75)	23	225.55 (40.46)	0.97	43	57.49 (32.09)	38	68.36 (70.93)	0.97
Reticulocytes, %	20	4.39 (1.01)	42	4.65 (1.29)	0.21	28	4.90 (1.82)	23	4.96 (1.37)	0.97	37	1.67 (2.70)	38	5.47 (18.50)	0.84
MCVr, fL	20	72.48 (3.49)	41	75.41 (4.93)	0.05	28	74.44 (3.94)	23	73.35 (4.45)	0.51	37	88.98 (7.43)	38	87.76 (8.14)	0.58
RDWr, %	20	14.95 (0.75)	41	14.75 (0.88)	0.57	28	14.65 (1.23)	23	14.51 (0.94)	0.89	37	13.79 (3.80)	38	12.78 (5.89)	0.39
CHCMr, mmol/L	20	15.13 (0.39)	41	14.99 (0.40)	0.35	28	14.55 (0.41)	23	14.68 (0.37)	0.24	37	13.42 (1.14)	38	13.06 (1.53)	0.26
HDWr, mmol/L	20	2.18 (0.36)	41	2.15 (0.29)	0.74	28	2.30 (0.42)	23	2.26 (0.23)	0.93	37	2.11 (0.76)	38	1.90 (0.89)	0.53
CHr, fmol	20	1.09 (0.06)	41	1.12 (0.07)	0.14	28	1.07 (0.05)	23	1.07 (0.05)	0.93	37	1.18 (0.11)	38	1.13 (0.10)	0.05

*SD* standard deviation *FeC* control group, *FeT* iron treated group, *HbC* hemoglobin concentration, *RBC* red blood cell, *Hct* hematocrit, *MCV* mean cell volume, *MCH* mean cell hemoglobin, *MCHC* mean cell hemoglobin concentration, *RDW* red blood cell distribution width, *HDW* hemoglobin distribution width, *MCVr* mean reticulocyte cellular volume, *RDWr* reticulocyte red cell distribution width, *CHCMr* mean reticulocyte cell hemoglobin concentration, *HDWr* reticulocyte hemoglobin distribution width, *Chr* reticulocyte hemoglobin content

### Tolerability of iron treatment

No obvious swelling, redness or lesions was seen in the injected sites in any of the sows. The farmer did not report any disorder related to the injections. All pregnancies were carried to normal term.

## Discussion

This study investigated the effects of an injected iron during gestation on sow and piglet hematology. The tolerability of the injected iron was also investigated. The injected iron did neither alter the sow hematology nor the piglet hematology. Similarly, no reduction in the probability of stillbirth was seen. The sows, tolerated well to the iron product at the given high dose. Absence of obvious swellings and lesions at the injection sites suggest no major injury to the tissues. None of the injected sows aborted or showed abnormal reproductive variables at farrowing.

The lack of effects on sow and piglet hematology may have several explanations. First, it should be borne in mind that the alteration in HbC and other hematologic variables are not only caused by low iron stores but also due to increased blood volume during pregnancy. The plasma volume in sows increases by up to 20% during pregnancy [22]. The sow herd had a good production level and no clinical signs of anemia although the average HbC was lower than four other sampled herds. Around half of the sows we studied were anemic based on HbC while none of the sows had microcytic erythrocytes. Although there were few sows (16%) with hypochromic erythrocytes at baseline, this change is probably physiological as the MCHC values rose up close to weaning. Also in pregnant women, microcytic anemia is not generally seen in pregnancy despite mild iron deficiency as the volume of RBC increases as a result of compensatory mechanism [23]. The sows in our study probably were not iron deficient enough to present microcytic erythrocytes and to respond to treatment. The average baseline HbC in FeT and FeC sows were within the reference limits for sows at mid-gestation [21]. A study in piglets has shown that animals with high HbC (> 100 g/L for a day old piglet) respond less to treatment [24]. If the sows in fact received iron according to the demands of the body by their standard feeds, this may have resulted in adequate iron absorption and utilisation irrespectively of treatment [25]. There were batch differences in most of the hematologic variables studied. The two batches of sows had a different course of HbC development during gestation and after farrowing, despite no obvious differences in feeding or management. The course of HbC development in batch 2 was as expected [18] i.e., a fall towards the end of gestation and a slight rise after farrowing. Batch 1 sows showed an opposite trend, i.e. an increase towards end of gestation and a fall after farrowing. The differences in blood variables in finishing pigs arising from different batches of the same herd have been reported earlier [26] and our study shows this phenomenon in gestating sows.

Secondly, we used a dose of iron that was higher than that used in other studies [9, 11] as these studies did not show effect of iron injection. It is possible that the dose of injected iron was high enough to stimulate hepcidin production. Hepcidin is the central regulatory hormone that down regulates iron absorption, macrophage iron release [27] and probably placental transfer of iron in the presence of high amounts of iron as seen in cows [28]. In piglets, it has been shown that high doses of iron injection may stimulate hepcidin synthesis, which hinders both the utilization of iron deposited in reticuloendothelial cells and the absorption of iron in feed [27]. In piglets receiving low doses of iron, the iron is mainly transported to the bone marrow for erythropoiesis, whereas in piglets receiving high doses of iron, the iron is partially transferred to the liver, where it induces hepcidin synthesis [27]. No such study has been made in sows. In this study, an iron preparation approved for piglets was used. The injected iron should perhaps have been divided into smaller split doses in order to reduce hepcidin synthesis, however, hepcidin levels were not measured in our study. It is quite impractical to provide split dose intramuscular injections in sow herds because of time and economic reasons. Oral supplementation or subcutaneous injections might be other strategies which allow slower release of iron. We used two split doses of iron injections for three reasons; to reduce tissue discomfort caused by large doses of iron, to reduce the risk of hepcidin stimulation and to supply extra iron to the sows when the growth of foetuses in the uterus is high.

Third, it is difficult to say how the injected iron distributed. There might be possibilities of high iron stores in the liver and other organs of the treated group sows which we could not determine. Determination of tissue iron levels combined with histopathologic evaluation would have been ideal to provide a more complete picture of iron homeostasis in sows and their offspring. Such techniques are laborious and difficult to perform in live animals. Frequent monitoring of the blood variables in the sows might provide insights into the changes brought by the treatments to better understand the metabolism of iron during mid-gestation.

TIBC and TSAT are important biochemical indicators of iron status in piglets [29, 30] and also probably in sows. FeC sows in this study had higher SI concentrations compared to FeT after treatment. SI is influenced by different factors such as feed intake and it shows diurnal variations of about 50% [31]. All samples were taken in the mornings to avoid possible diurnal variations but alterations due to feed intake could not be avoided. TIBC was higher in the FeC animals as expected as it is the maximum amount of iron that can bind with transferrin. It is surprising that there is a discrepancy in SI and TIBC concentrations as SI was expected to be increased in FeT group, but in fact it was decreased. As SI and TIBC are measures of iron transportation but not stores, they are not reliable in evaluating physiologic needs of iron in the pregnant sows. However, the SI and TIBC concentrations in both the groups after interventions were within the reference limits for sows [32]. Studies suggest that using TSAT values instead of serum iron or TIBC separately provides a reliable estimate of the iron status in animals [33]. In this study, TSAT was similar in both the groups indicating similar iron status. The mean concentrations of TSAT in both the groups were slightly higher than the upper reference limits calculated from a previous study [32]. Serum ferritin, which is regarded as very reliable to diagnose iron deficiency and iron deficiency anemia in human medicine [34] was however not analysed in this study. It should also be noted that there was some variation in blood sampling days because all sows belonging to the same week batch were sampled on the same day for practical reasons and these measurements were assigned to the same time point during comparison. This might be a source of variation in hematologic results.

An earlier study in Denmark [11] had found no effect of iron injection (1600 mg) 3 weeks before farrowing on sow HbC. The sows in that study had a higher mean HbC baseline level (124 g/L) compared to our study. Another study [9] did not find an effect of 1000 mg of injected iron 2 weeks before farrowing neither on sow HbC nor in the number of stillborn piglets. Also no significant improvement in the number of liveborn piglets was found when sows were injected with 1600 mg iron 3 weeks before farrowing [35]. A number of other studies have shown the absence of effects after different dietary supply of iron when sows were fed iron diet at different stages of gestation [8, 10, 36]. A study in small number of sows (n = 10) demonstrated improved litter size and litter weight when sows were fed high iron diet (256 mg/kg) during entire gestation [7]; nevertheless, blood variables in the sows did not differ between the groups. It is worth mentioning that these studies used different forms of iron and the bioavailability of iron is largely dependent on its type [37]. All these studies suggest that improving the hematology in sows by iron treatment is difficult and requires additional insight in the factors controlling erythropoiesis in sows. However, it should be noted that these studies mainly included sows without well-defined anemia.

The piglets born from the two experimental groups did not differ significantly in body weight and hematologic variables. The probable reason might be very little iron transfer through the placenta in swine. It has been shown that the amount of iron transferred from sow to the fetus is very low compared to the other mammals [38], and this may be the main reason why piglets are born with very low iron reserves irrespective of maternal iron status.

The stillbirth rate in both batches was lower compared to the herd average, which could be related to increased awareness of the farm personnel regarding the trial. This might also be one of the reasons for lack of effect of iron injection on probability of stillbirth. Stillbirth in sows is caused by a number of factors including sow, piglet and other environmental factors [39]. Some of those factors could not be controlled in our study. Therefore, future studies on pregnant sows with well-defined iron deficiency anemia including other factors causing stillbirths should be considered.

## Conclusions

Iron treatment of mid-gestating sows did neither change hematologic variables in sows nor in the piglets at farrowing. Stillbirth rate was not influenced by the treatment. Although anemia was prevalent in the sows before treatment, no indications of specific iron deficiency anaemia were observed which may explain the lack of treatment effect. Gestational physiology of sows in relation to iron stores has not been studied extensively and further research is needed in order to improve HbC in sows during gestation.

## Data Availability

The datasets used and/or analysed during the current study are available from the corresponding author on reasonable request.

## Abbreviations

CHCMr: mean reticulocyte cell hemoglobin concentration
Chr: reticulocyte hemoglobin content
EDTA: ethylenediamine tetraacetic acid
FeT: treatment
FeC: control
Hb: hemoglobin
HbC: hemoglobin concentration
HCT: hematocrit
HDW: hemoglobin distribution width
HDWr: reticulocyte hemoglobin distribution width
IDA: iron deficiency anemia
MCH: mean cell hemoglobin
MCHC: mean cell hemoglobin concentration
MCV: mean cell volume
MCVr: mean reticulocyte cellular volume
RBC: red blood cells/erythrocytes
RDW: red blood cell distribution width
RDWr: reticulocyte red cell distribution width
SI: serum iron
SPF: specific pathogen free
TIBC: total iron binding capacity
TSAT: transferrin saturation
